# Feedback Regulations of miR-21 and MAPKs via Pdcd4 and Spry1 Are Involved in Arsenite-Induced Cell Malignant Transformation

**DOI:** 10.1371/journal.pone.0057652

**Published:** 2013-03-01

**Authors:** Lu Shen, Min Ling, Yuan Li, Yuan Xu, Yun Zhou, Jing Ye, Ying Pang, Yue Zhao, Rongrong Jiang, Jianping Zhang, Qizhan Liu

**Affiliations:** 1 Institute of Toxicology, Ministry of Education, School of Public Health, Nanjing, Jiangsu, People’s Republic of China; 2 The Key Laboratory of Modern Toxicology, Ministry of Education, School of Public Health, Nanjing, Jiangsu, People’s Republic of China; 3 Department of General Surgery, the Second Affiliated Hospital, Nanjing Medical University, Nanjing, Jiangsu, People’s Republic of China; 4 Jiangsu Center for Disease Control and Prevention, Nanjing, Jiangsu, People’s Republic of China; University of Pittsburgh Cancer Institute, United States of America

## Abstract

**Objective:**

To establish the functions of miR-21 and the roles of two feedback regulation loops, miR-21-Spry1-ERK/NF-κB and miR-21-Pdcd4-JNK/c-Jun, in arsenite-transformed human embryo lung fibroblast (HELF) cells.

**Methods:**

For arsenite-transformed HELF cells, apoptosis, clonogenicity, and capacity for migration were determined by Hoechst staining, assessment of their capacity for anchorage-independent growth, and wound-healing, respectively, after blockage, with inhibitors or with siRNAs, of signal pathways for JNK/c-Jun or ERK/NF-κB. Decreases of miR-21 levels were determined with anti-miR-21, and the up-regulation of Pdcd4 and Spry1 was assessed in transfected cells; these cells were molecularly characterized by RT-PCR, qRT-PCR, Western blots, and immunofluorescence assays.

**Results:**

MiR-21 was highly expressed in arsenite-transformed HELF cells and normal HELF cells acutely treated with arsenite, an effect that was concomitant with activation of JNK/c-Jun and ERK/NF-κB and down-regulation of Pdcd4 and Spry1 protein levels. However, there were no significant changes in mRNA levels for Pdcd4 and Spry1, which suggested that miR-21 regulates the expressions of Pdcd4 and Spry1 through translational repression. In arsenite-transformed HELF cells, blockages of JNK/c-Jun or ERK/NF-κB with inhibitors or with siRNAs prevented the increases of miR-21and the decreases of the protein levels but not the mRNA levels of Pdcd4 and Spry1. Down-regulation of miR-21 and up-regulations of Pdcd44 or Spry1 blocked the arsenite-induced activations of JNK/c-Jun or ERK/NF-κB, indicating that knockdown of miR-21 inhibits feedback of ERK activation and JNK activation via increases of Pdcd4 and Spry1 protein levels, respectively. Moreover, in arsenite-transformed HELF cells, inhibition of miR-21 promoted cell apoptosis, inhibited clonogenicity, and reduced migration.

**Conclusion:**

The results indicate that miR-21 is both a target and a regulator of ERK/NF-κB and JNK/c-Jun and the feedback regulations of miR-21 and MAPKs via Pdcd4 and Spry1, respectively, are involved in arsenite-induced malignant transformation of HELF cells.

## Introduction

Chronic exposure to arsenite induces cellular transformation characterized by increased proliferation and anchorage-independent growth [1], [2]. Arsenite has effects on activation of signal pathways, such as mitogen-activated protein kinases (MAPKs), phosphoinositide-3-kinase (PI-3K)/Akt (also known as protein kinase B), and nuclear factor-κB (NF-κB) [3], [4]. Although skin is thought to be the most sensitive tissue for arsenic toxicity, lung is now recognized as a target as well [5], [6]. Even though multiple hypotheses have been proposed to explain arsenite-induced carcinogenesis, the exact mechanism remains elusive.

MicroRNAs (miRNAs), small, non-coding RNA molecules of 21 to 23 nucleotides, have the capacity to inhibit translation and induce mRNA degradation, predominantly through the 3′-untranslated regions (3′-UTR) of mRNAs [7]. The involvement of miRNAs in lung carcinogenesis has yet to be explored [8]. MicroRNA-21 (miR-21) is over-expressed in carcinomas of lung, prostate, breast, pancreas, colon, head and neck, stomach, esophagus, and liver, relative to adjacent normal tissues, supporting the concept that miR-21 is a ubiquitous oncogene [9], [10]. Moreover, miR-21 is implicated in various processes associated with malignant transformation, such as cell proliferation, apoptosis, invasion, and metastasis [11], [12]. Although our previous studies showed that reactive oxygen species-activated miR-21-Spry1-ERK/NF-κB loop regulation is involved in arsenite-induced cell transformation of human embryo lung fibroblast (HELF) cells [13], the roles of miR-21 in arsenite-transformed cells is unknown.

Programmed cell death protein 4 (Pdcd 4) is a tumor suppressor that is down-regulated or absent in various tumors [14], [15]. Its ectopic expression reduces tumor formation, inhibits cellular invasion, and promotes cell apoptosis [16], [17]. MiR-21 is a negative regulator of Pdcd4, and Pdcd4 likely contributes to miR-21-induced tumor cell invasion and anti-apoptosis [18], [19]. Furthermore, Pdcd4 blocks c-Jun activation by inhibiting the expression of mitogen-activated protein kinase kinase kinase kinase 1 (MAP4K1) (also known as hematopoietic progenitor kinase 1), which is up-stream of Jun N-terminal kinase (JNK) [20], [21]. The c-Jun-interacting region of the miR-21 promoter has been identified [19], [22], and the migration and invasion promoted by the miR-21-Pdcd4-JNK/c-Jun feedback loop has been confirmed in human tumors [22], [23], [24]. Therefore, we postulated that the miR-21-Pdcd4-JNK/c-Jun feedback loop is involved in arsenite-induced cell transformation.

The mammalian Spry family has four members (Spry1–4), which differ in tissue distribution, activity, and interaction partners [25]. Expressions of Spry genes, specially Spry1 and Spry2 isoforms, are frequently decreased or absent in human cancers, implicating them as suppressors of tumorigenesis [26], [27]. In general, Spry1 and Spry2 negatively regulate growth factor-induced cellular proliferation, migration, and differentiation [28]. The levels of Spry1 and Spry2 are controlled by miR-21 [8], [29], however, we previously found that the inhibition of miR-21 prevents arsenite-induced decreases in Spry1 but not Spry2 in HELF cells [13]. Spry1 has been proposed to function as a tumor-suppressor gene in various malignancies, including prostate cancer and hepatocellular carcinoma [30]. The down-regulation of Spry1 is essential for the maximal induction of extracellular signal-regulated kinase (ERK) activity [31]. Spry1 specifically inhibits receptor tyrosine kinase (RTK)-mediated Ras-ERK/MAPKs signaling [32].

In the present effort, the roles of miR-21 and two feedback regulation loops, miR-21-Spry1-ERK/NF-κB and miR-21-Pdcd4-JNK/c-Jun, were investigated in arsenite-transformed HELF cells. The results, pointing to miR-21 as both a target and a regulator of ERK/NF-κB and JNK/c-Jun, reveal a novel auto-regulatory loop mediated by miR-21 and Pdcd4. The results provide evidence that miR-21 reduces the expression of its target genes to exert an oncogenic function.

## Materials and Methods

### Cell Culture

Immortalized normal HELF (HELF-C) cells were obtained from the Shanghai Institute of Cell Biology, Chinese Academy of Sciences (Shanghai, China). HELF cells are human sarcoma virus (SV)–40 immortalized, non-tumorigenic, diploid fibroblasts from the lungs of hysterotomy-derived embryos [33]. HELF cells have normal signal pathways and are used as a model of lung damage and neoplastic transformation induced by environmental agents [34], [35]. As previously reported, normal HELF cells exposed to 0.0 or 1.0 µM arsenite for 30 passages (about 15 weeks) are used as the passage-control HELF (HELF-30C) cells and arsenite-transformed HELF (HELF-30T) cells, respectively [13], [36]. Cells were maintained in 5% CO_2_ at 37°C in Dulbecco’s Modified Eagle Medium (DMEM, Life Technologies/Gibco, Grand Island, NY) supplemented with 10% fetal bovine serum (FBS, Life Technologies/Gibco), penicillin (100 U/ml), and streptomycin (100 µg/ml, Life Technologies/Gibco, Gaithersburg, MD). For chronic exposure, 1×10^6^ cells were seeded into 10-cm (diameter) dishes for 24 h and maintained in 0.0 or 1.0 µM arsenite (NaAsO_2_; Sigma, St. Louis, MO, USA; 99.0% purity) for 48–72 h per passage. This process was continued for about 15 weeks (30 passages).

### Cell Transfection

Control siRNA, NF-κB p65 siRNA, and c-Jun siRNA were purchased from Cell Signaling Technology. Transfections were performed with the N-TERTM Nanoparticle siRNA Transfection System (Sigma). Anti-miR-21 and miRNA NC (negative control) were synthesized by RiBoBio (Guangzhou, China). Cells were transiently transfected by use of the Lipofectamine 2000 reagent (Invitrogen, Carlsbad, CA) according to the manufacturer’s protocol. At 24 h after transfection, cells were harvested for use in experiments.

### Transient Transfection Assay

The plasmids of pIRES-Pdcd4 construct and pIRES-Spry1 construct were purchased from Generay Biotechnology (Shanghai). The arsenite-transformed HELF cells were transiently transfected using the Lipofectamine 2000 reagent (Invitrogen, Carlsbad, CA, USA) according to the manufacturer’s protocol. At 24 h after transfection, cells were harvested and used for experiments.

### Apoptosis Assay

Cells were placed in 6-well plates and transfected as described. At 24 h after transfection and 4 h of serum starvation, the cells in each well were washed twice with cold, phosphate-buffered saline (PBS) and fixed with 4% (v/v) formaldehyde in PBS for 10 min at room temperature. The cells were washed twice with PBS and stained with 10 µg/mL Hoechst 33258 (Sigma, Louis, MO, USA) in PBS at 37°C for 20 min, and then examined under a fluorescent microscope for morphological changes consistent with apoptotic cell death. Apoptotic cells were counted using ImageJ software (http://rsb.info.nih.gov/ij/).

### Anchorage-independent Growth Assay

Soft-agar dishes were prepared with under-layers of 0.70% agarose in DMEM medium supplemented with 10% FBS. To test for colony growth capacity, cells were plated at a density of 1×104 in 1 ml of 0.35% agarose over the agar base. Cultures were fed every three days with DMEM medium supplemented with 10% FBS, and, after for 14 days, colonies with >30 cells were examined microscopically.

### Wound-healing Assay

Cell migration was measured by the wound healing assay, as previously described [21], [52]. Cells (7×10^5^ cells per well) were seeded in six-well plates and serum-starved for 24 h, after which the medium was replaced (10% FBS). Wounds were made by passing a plastic tip across the monolayer cells. Wound infliction was considered as 0 h, and wound closure was monitored for up to 24 h. Wound closures were photographed by phase contrast microscopy (40X) at 12 h after scraping. The width of the wound was determined with the Image Pro-Plus program.

### RNA Extraction and Semiquantitative RT-PCR

Total RNA from passage control HELF cells or arsenite-transformed HELF cells subjected to different transfection protocols was extracted with Tri reagent (Molecular Research Center) following the manufacturer’s instructions. The primers for Pdcd4 were as follows: forward, *5′-ATGGATATAGAAAATGAGCAGAC-3′,* and reverse, *5′-CCAGATCTGGACCGCCTATC-3′*. The primers for *spry1* amplification were: sense primer, *5′-GTGTGTTGGAAATCCACGGT-3′,* and antisense primer, *5′-AAAGAAGGCTGCTGGATCAC-3′*. Amplified glyceraldehyde 3-phosphate dehydrogenase (GAPDH) was used as housekeeping gene. Primers for GAPDH were: sense, *5′-TCCCATCACCATCTTCCA-3′*, and antisense, *5′-CATCACGCCACAGTTTCC-3′*. For each gene, the number of cycles was optimized to fall within the linear range of PCR amplification. PCR products were resolved on 1.5% (wt/vol) agarose gels containing ethidium bromide. Gel images were digitally recorded, and amplicon levels were quantified by the computer-assisted image analyzer, Gel-Pro (IPS, North Reading, MA, USA).

### Quantitative Reverse Transcription (qRT)-PCR

Total cellular RNA was isolated by use of Trizol (Invitrogen, Carlsbad, CA) according to the manufacturer’s recommendations. For detection of mature miR-21, 2 µg of total RNA, miRNA-specific stem–loop reverse transcription (RT) primers and MMLV reverse transcriptase (Promega Corporation, Madison, WI) were used in a reverse transcription following the manufacturer’s protocol. The RT primers for miR-21 and U6 small nuclear RNA (snRNA) were as follows, miR-21: 5′-*CTCAACTGGTGTCGTGGAGTCGGCAATTCAGTTGAGTCAACATC*-3′, and U6 snRNA, 5′-*AAAATATGGAACGCTTCACG*-3′. The sequences of mature miRNAs were from Sanger miRBase (http://microrna.sanger.ac.uk/sequences/). qRT-PCR was performed with Power SYBR Green Master Mix (Applied Biosystems, Foster City, CA) and an ABI 7300 real-time PCR detection system (Applied Biosystems). Forward (F) and reverse (R) primers were as follows: miR-21-F, 5′-*ACACTCCAGCTGGGTAGCTTATCAGACTGA*-3′; miR-21-R: 5′-*TGGTGTCGTGGAGTCG*-3′; U6-F, 5′-*CGCTTCGGCAGCACATATACTAAAATTGGAAC*-3′; and U6-R, 5′-*GCTTCACGAATTTGCGTGTCATCCT TGC*-3′. All of the primers were synthesized by Invitrogen. U6 snRNA was used as an internal control. Fold changes in expression of each gene were calculated by a comparative threshold cycle (Ct) method using the formula: 2^−(ΔΔCt)^.

### Western Blots

Cell lysates were separated by sodium dodecyl sulfate-polyacrylamide gel electrophoresis and were transferred to polyvinylidene fluoride membranes (Millipore, Billerica, MA, USA); the immune complexes were detected by enhanced chemiluminescence (Cell Signaling Technology, Beverly, MA, USA). Antibodies used were those for ERK, p-ERK (Thr 202/Tyr 204), JNK, p-JNK (Thr183/Tyr185), p38, p-p38 (Thr 180/Tyr 182), NF-κB p65, p-NF-κB p65 (Ser 536), c-Jun, p-c-Jun (Ser 63), Akt, p-Akt (Ser473), Pdcd4, Spry1, cleaved-caspase-3, and caspase-3 (Cell Signaling Technology); and GAPDH (Sigma). Densities of bands were quantified by Eagle Eye II software. GAPDH levels, measured in parallel, served as controls.

### Statistics

All statistical analyses were accomplished with SPSS for Windows, version 13.0. All numeric data were generated from three independent experiments and expressed as means ± SD. One-way analysis of variance (ANOVA) was used to assess differences among groups. Statistical significance, determined by the Fisher test, was set at *P*<0.05.

## Results

### The Activations of ERK/NF-κB, JNK/c-Jun, and Akt are Induced by Arsenite in HELF Cells

**Figure 1:**
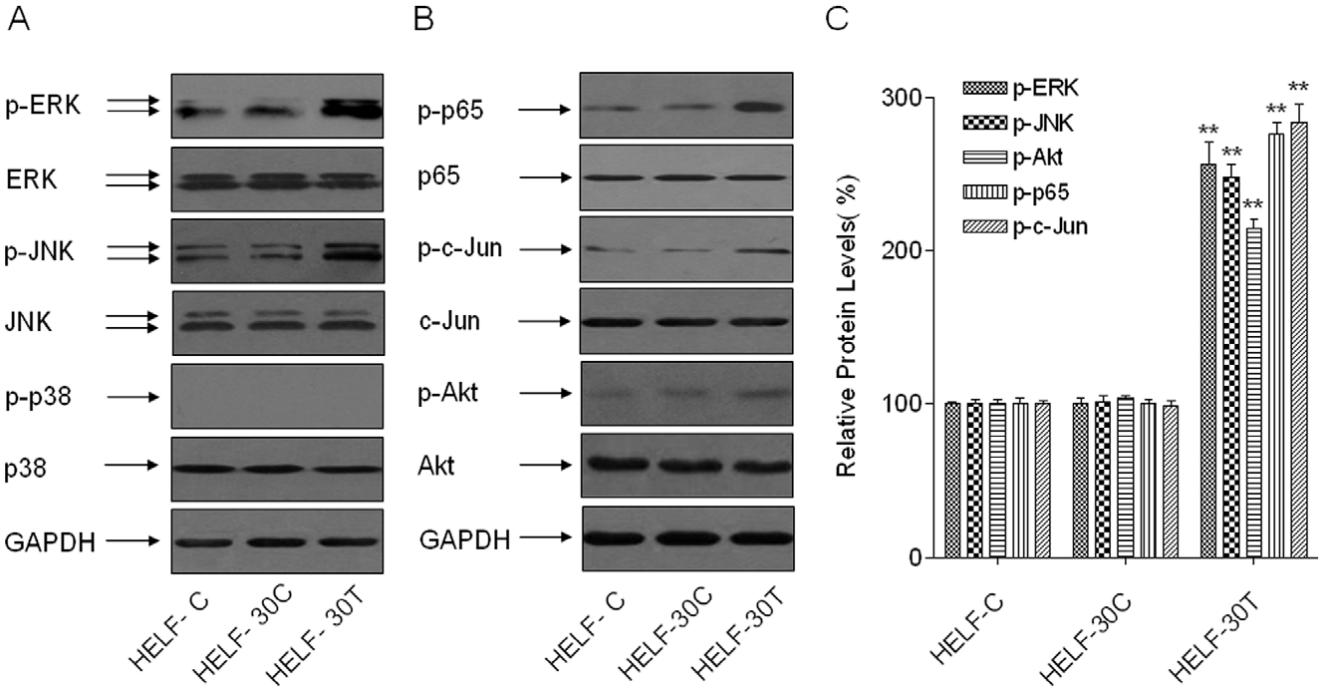
Activations of ERK/NF-κB, JNK/c-Jun, and Akt are induced in arsenite-transformed HELF cells. Abbreviations: HELF-C, normal HELF cells; HELF-30C, passage-control HELF cells; HELF-30T, arsenite-transformed HELF cells. Densities of bands were quantified by Eagle Eye II software. GAPDH levels, measured in parallel, served as controls. HELF cells were exposed to 0.0 or 1.0 µM arsenite for 30 passages. (A, B) Western blot analyses and (C) relative protein levels (means ± SD, n = 3) of p-ERK, p-JNK, and p-p38 (representative of MAPKs signal pathways); p- NF-κB 65 and p-c-Jun levels (representative transcription factors); and p-Akt level (representative of the PI-3Ks signal pathway). **P<0.01 different from HELF-30C cells.

To determine whether the MAPKs and PI-3Ks signal pathways are involved in arsenite-induced malignant transformation of HELF cells, the levels of p-ERK, p-JNK, and p-p38 (representative of MAPKs signal pathways); the levels of p-NF-κB p65 and p-c-Jun (representative transcription factors); and the level of p-Akt (representative of the PI-3Ks signal pathway) were determined in normal HELF cells, in passage-control HELF cells, in arsenite-transformed HELF cells, and in normal HELF cells treated with 1.0 µM arsenite for 0, 6, or 24 h. The levels of p-ERK, p-JNK, p-Akt, p-NF-κB-p65, and p-c-Jun were increased in arsenite-transformed HELF cells, compared with normal HELF cells and passage-control HELF cells. However, the levels of p-p38 were not induced significantly (Figure 1A, 1B, and 1C). These results are consistent with acute exposure of normal HELF cells to 1.0 µM arsenite (Figure S1). Thus, the activations of ERK/NF-κB and JNK/c-Jun are associated with arsenite-induced malignant transformation of HELF cells.

### The Level of miR-21 is Up-regulated, and the Protein Levels of Pdcd4 and Spry1 are Decreased by Arsenite in HELF Cells

**Figure 2:**
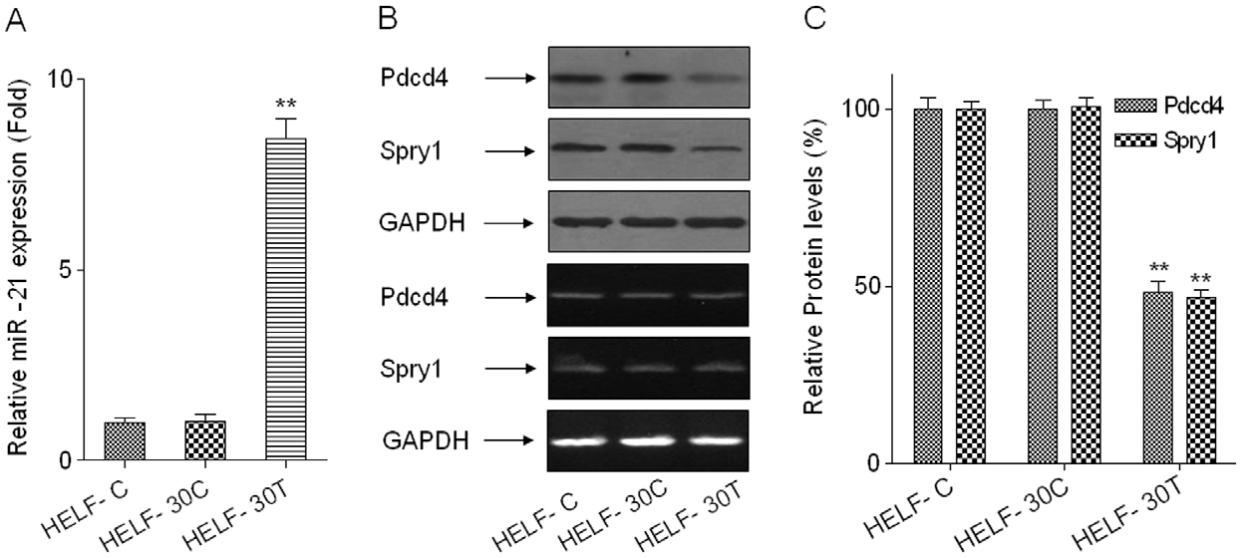
The level of miR-21 is up-regulated, and the protein levels of Pdcd4 and Spry1 are decreased in arsenite-transformed HELF cells. Abbreviations: HELF-C, normal HELF cells; HELF-30C, passage-control HELF cells; HELF-30T, arsenite-transformed HELF cells. Densities of bands were quantified by Eagle Eye II software. GAPDH levels, measured in parallel, served as controls. HELF cells were exposed to 0.0 or 1.0 µM arsenite for 30 passages. (A) The levels of miR-21 were determined by qRT-PCR assays (means ± SD, n = 3). **P<0.01 different from HELF-30C cells. (B) The protein levels (upper) and mRNA levels (lower) of Pdcd4 and Spry1 (target proteins of miR-21) were analyzed by Western blots and RT-PCR, respectively. (C) The relative protein levels of Pdcd4 and Spry1 (means ± SD, n = 3). **P<0.01 different from HELF-30C cells.

Over-regulation of miR-21 is implicated in malignancy-related processes, including cell proliferation, apoptosis, invasion, and metastasis [3], [11]. To determine if miR-21 is involved in arsenite-induced transformation of HELF cells, miR-21 expression and the protein levels and miRNA levels of Pdcd4 and Spry1, target genes of miR-21, were investigated in normal HELF cells, in passage-control HELF cells, in arsenite-transformed HELF cells, and in normal HELF cells treated with 1.0 µM arsenite for 0, 6, or 24 h. The level of miR-21 was 8.8-fold higher in arsenite-transformed cells relative to passage-control cells (Figure 2A). In arsenite-transformed HELF cells, the protein levels of Pdcd4 and Spry1 were lower than those in passage control cells; however, there were no significant differences for mRNA levels of Pdcd4 and Spry1 in two groups of cells (Figure 2B and 2C). The results were the same for normal HELF cells treated acutely with 1.0 µM arsenite (Figure S2). Thus, over-expression of miR-21 and the decreases of target proteins are associated with arsenite-induced malignant transformation.

#### ERK/NF-κB and JNK/c-Jun are involved in the regulations of miR-21 to protein levels of Pdcd4 and Spry1 and to cell apoptosis in arsenite-transformed HELF cells

**Figure 3:**
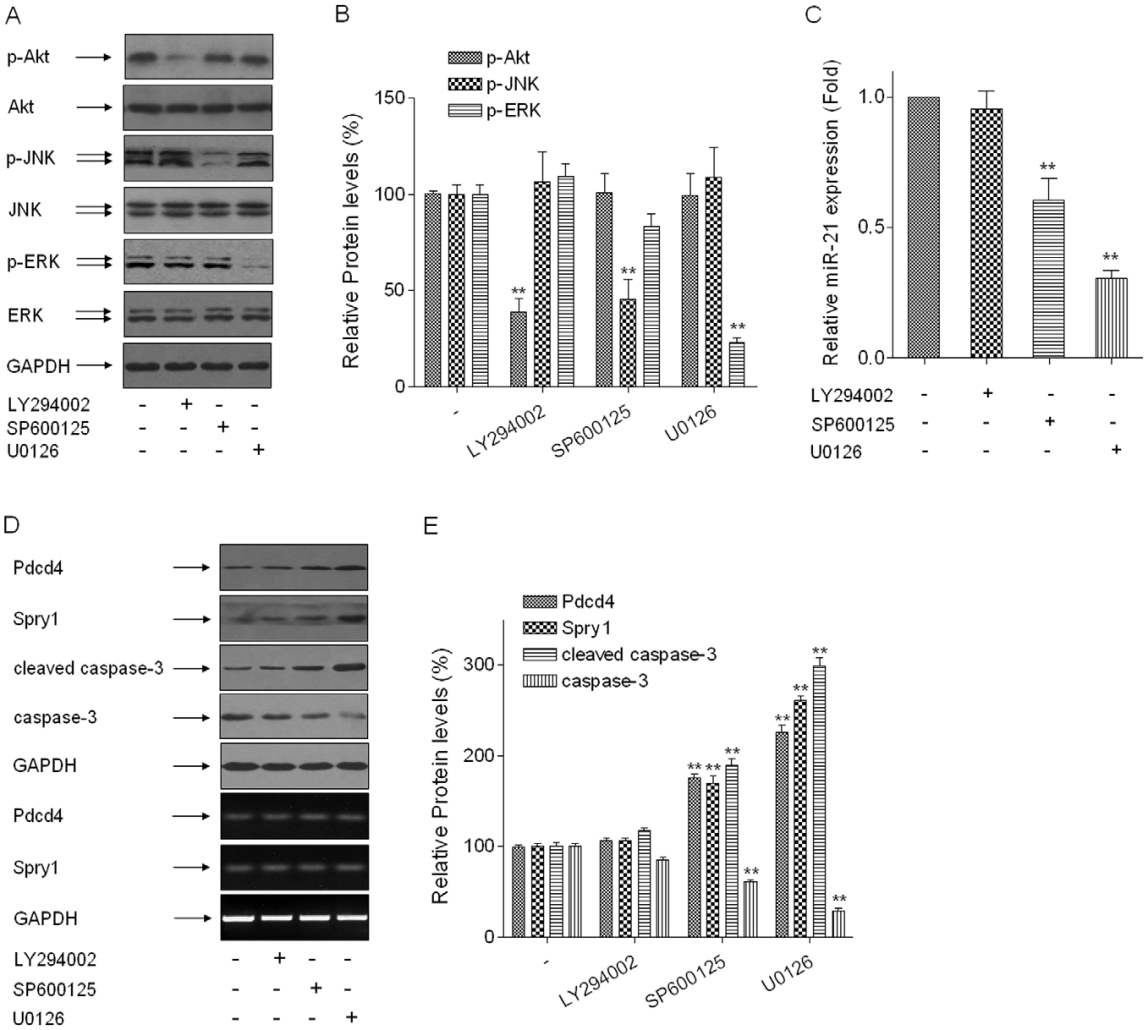
ERK and JNK are involved in the regulations of miR-21 to protein levels of Pdcd4 and Spry1 and to cell apoptosis in arsenite-transformed HELF cells. Densities of bands were quantified by Eagle Eye II software. GAPDH levels, measured in parallel, served as controls. Arsenite-transformed HELF cells were treated with 10 µM of LY294002 (an inhibitor of PI-3K), SP600125 (an inhibitor of JNK), or U0126 (an inhibitor of ERK) for 24 h. (A) Western blots and (B) relative protein levels (means ± SD, n = 3) of p-Akt, p-ERK, and p-JNK. (C) qRT-PCR analysis of miR-21 expression (means ± SD, n = 3). **P<0.01 difference from control group. (D) Western blots (upper) and RT-PCR (below) analyses for protein and mRNA levels and (E) relative protein levels (means ± SD, n = 3) of Pdcd4, Spry1, cleaved-caspase-3, and caspase-3. **P<0.01 difference from control group.

To elucidate how arsenite regulates miR-21 and its target genes, arsenite-transformed HELF cells were treated with 10 µM of LY294002 (an inhibitor of PI-3K), SP600125 (an inhibitor of JNK), or U0126 (an inhibitor of ERK). Inhibition of ERK or JNK by SP600125 or U0126 blocked the arsenite-induced increases of miR-21 expression; however, inhibition of PI-3K by LY294002 caused no obvious change in miR-21 expression (Figure 3A, 3B, and 3C). The reduction of miR-21 by blockage of JNK or ERK increased the protein levels but not the mRNA levels of Pdcd4 and Spry1. The inhibition of JNK or ERK by SP600125 or U0126 induced increases of cleaved-caspase-3 levels and decreases of caspase-3 levels (Figure 3D and 3E).

**Figure 4:**
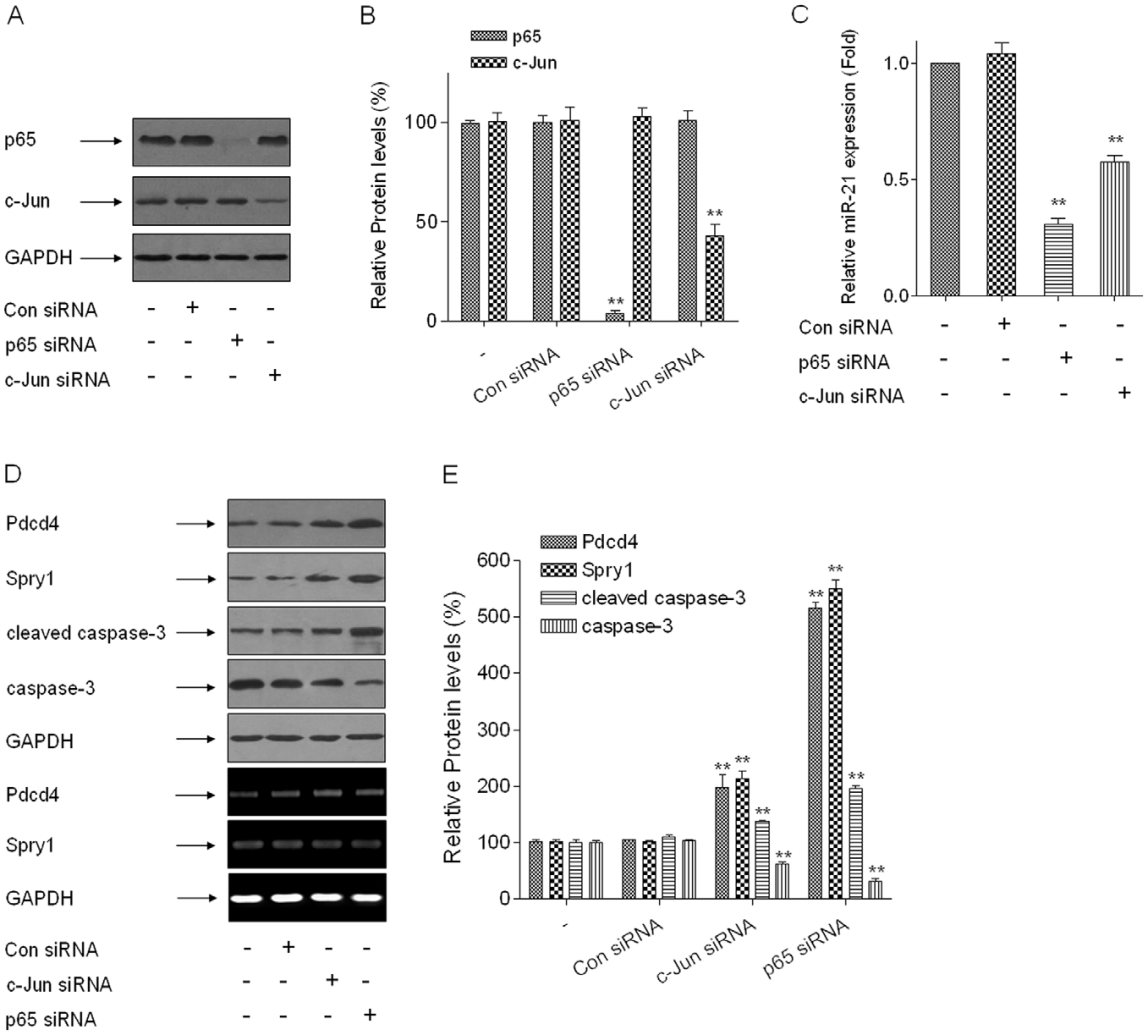
NF-κB and c-Jun are involved in the regulations of miR-21 to protein levels of Pdcd4 and Spry1 and to cell apoptosis in arsenite-transformed HELF cells. Densities of bands were quantified by Eagle Eye II software. GAPDH levels, measured in parallel, served as controls. Arsenite-transformed HELF cells were transfected with 50 nM of control siRNA, NF-κB p65 siRNA, or c-Jun siRNA for 24 h. (A) Western blots and (B) relative protein levels (means ± SD, n = 3) of NF-κB p65 and c-Jun. (C) qRT-PCR analysis of miR-21 expression (means ± SD, n = 3). **P<0.01 difference from Con siRNA group. (D) Western blots (upper) and RT-PCR (below) analyses for protein and mRNA levels and (E) relative protein levels (means ± SD, n = 3) of Pdcd4, Spry1, cleaved-caspase-3, and caspase-3, **P<0.01 difference from the control (Con) siRNA group.

Furthermore, siRNAs were applied to determine the effects of the transcriptional factors, NF-κB p65 and c-Jun, on miR-21 expression. Knockdown of NF-κB p65 or c-Jun attenuated the increases of miR-21 expression. NF-kB p65 knockdown caused an 80–90% decrease in miR-21, and c-Jun depletion resulted in a 35–45% decrease (Figure 4A, 4B, and 4C). The reduction of miR-21 by knockdown of NF-κB p65 or c-Jun increased the protein levels but not the mRNA levels of Pdcd4 and Spry1. The blockage of NF-κB p65 or c-Jun with siRNA induced increases of cleaved-caspase-3 levels and decreases of caspase-3 levels (Figure 4D and 4E). Our previous studies have revealed that, for HELF cells exposed to arsenite, NF-κB and c-Jun are activated by ERK and JNK, respectively [13], [36]. These results suggest that arsenite-induced increases of miR-21 are regulated by ERK and JNK through the activations of NF-κB p65 and c- Jun, which down-regulate protein levels of Pdcd4 and Spry1 and promote cell apoptosis.

#### Pdcd4 and Spry1 are target proteins of miR-21

**Figure 5:**
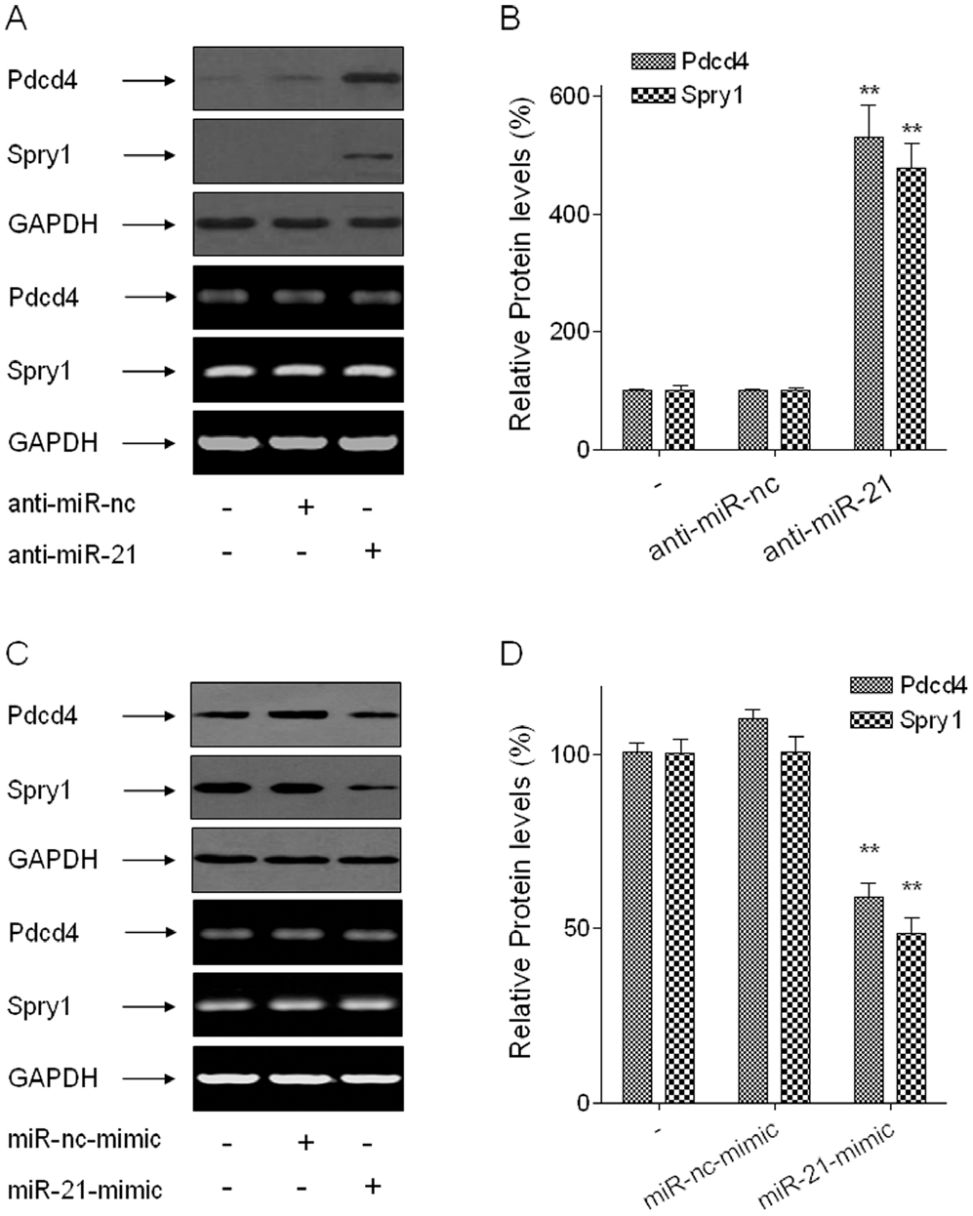
Pdcd4 and Spry1 are target proteins of miR-21. Densities of bands were quantified by Eagle Eye II software. GAPDH levels, measured in parallel, served as controls. Arsenite-transformed HELF cells were transfected with 150 nM of anti-miR-nc or anti-miR-21 for 24 h. (A) Western blots (upper) and RT-PCR (below) analyses for protein and mRNA levels of Pdcd4 and Spry1, respectively. (B) Relative protein levels of Pdcd4 and Spry1 (means ± SD, n = 3). **P<0.01 different from NC group. Normal HELF cells were transfected with 100 nM of miR-nc-mimic or miR-21-mimic for 24 h. (C) Western blots (upper) and RT-PCR (below) analyses for protein and mRNA levels of Pdcd4 and Spry1, respectively. (D) Relative protein levels of Pdcd4 and Spry1 (means ± SD, n = 3). **P<0.01 different from NC group.

To elucidate the relationship between the increases of miR-21 and the changes of Pdcd4 and Spry1 protein levels, the mRNA and protein levels of Pdcd4 and Spry1 were determined after the arsenite-transformed HELF cells were transfected with anti-miR-21 (150 nM) for 24 h. There were no changes in the Pdcd4 and Spry1 mRNA levels; however, inhibition of miR-21 elevated the protein levels of Pdcd4 and Spry1 (Figure 5A and 5B). Similarly, after normal HELF cells were transfected with an miR-21-mimic (100 nM) for 24 h, the increases of miR-21 decreased the protein levels but not the mRNA levels of Pdcd4 and Spry1 (Figure 5C and 5D), results that are consistent with previous studies indicating that miR-21 targets Pdcd4 and Spry1 [37], [38]. These findings demonstrate that Pdcd4 and Spry1 are regulated by miR-21.

#### The activations of ERK and JNK are feedback-regulated by miR-21 via Spry1 and Pdcd4, respectively, in arsenite-transformed HELF cells

**Figure 6:**
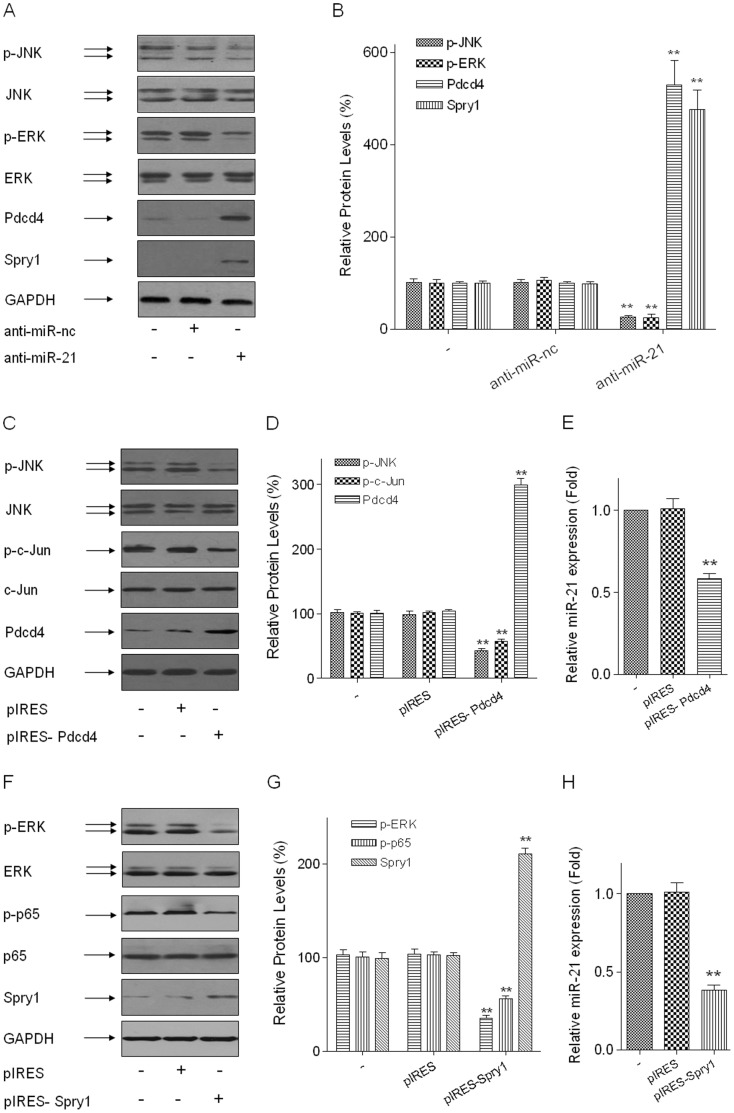
The activations of ERK and JNK are feedback-regulated by miR-21 via Spry1 and Pdcd4, respectively, in arsenite- transformed HELF cells. Densities of bands were quantified by Eagle Eye II software. GAPDH levels, measured in parallel, served as controls. Arsenite-transformed HELF cells were transfected with 150 nM of anti-miR-nc or anti-miR-21 for 24 h. (A) Western blots and (B) relative protein levels (means ± SD, n = 3) of p-ERK, p-JNK, Spry1, and Pdcd4. **P<0.01 different from NC group. Arsenite-transformed HELF cells were transfected with pIRES, pIRES-Pdcd4, or pIRES-Spry1 for 24 h. (C) Western blots and (D) relative protein levels (means ± SD, n = 3) of p-JNK, p-c-Jun, and Pdcd4. (E) qRT-PCR analysis of miR-21 expression (means ± SD, n = 3). **P<0.01 different from control group. (F) Western blots and (G) relative protein levels (means ± SD, n = 3) of p-ERK, p-NF-κB p65, and Spry1. (H) qRT-PCR analysis of miR-21 expression (means ± SD, n = 3). **P<0.01 different from control group.

For miR-21-mediated down-regulation of its target genes, Spry1 and Pdcd4, potent inhibitors of the ERK and JNK pathways are essential [8], [39]. To determine if the arsenite-induced activations of ERK and JNK are feedback-regulated by miR-21, arsenite-transformed HELF cells were transfected with anti-miR-21 (150 nM) for 24 h. For these cells, the protein levels of Spry1 and Pdcd4 were elevated, and the increases of p-ERK and p-JNK levels were blocked (Figure 6A and 6B). In arsenite-transformed HELF cells transiently transfected with pIERS-Pdcd4, up-regulation of Pdcd4 blocked the activations of JNK and c-Jun and increases of miR-21 expression (Figure 6C, 6D, and 6E). Similarly, the arsenite-induced activations of ERK and NF-κB and increases of miR-21 expression were blocked by up-regulation of Spry1 in arsenite-transformed HELF cells transiently transfected with pIRES-Spry1 (Figure 6F, 6G, and 6H). These results indicate that, in arsenite-transformed HELF cells, miR-21 regulates feedback on the activations of ERK and JNK via Spry1 and Pdcd4, respectively.

### Inhibition of miR-21 Increases Cell Apoptosis of Arsenite-transformed HELF Cells

**Figure 7:**
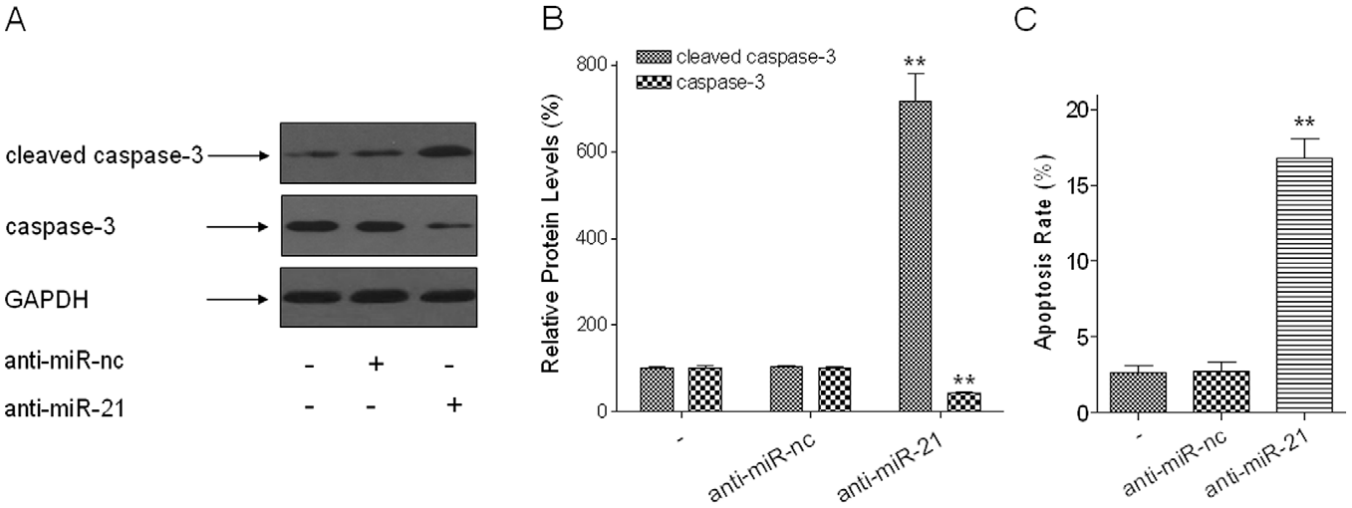
Inhibition of miR-21 increases cell apoptosis of arsenite-transformed HELF cells. Densities of bands were quantified by Eagle Eye II software. GAPDH levels, measured in parallel, served as controls. Arsenite-transformed HELF cells were transfected with 150 nM of anti-miR-nc or anti-miR-21 for 24 h. (A) Western blots and (B) relative protein levels of cleaved caspase-3 and caspase-3 (means ± SD, n = 3). **P<0.01 different from NC group. (C) The rate of cell apoptosis was analyzed by the Hoechst 33258 assay (means ± SD, n = 3). **P<0.01 different from NC group.

To determine if miR-21 functions as an onco-miRNA or as anti-onco-miRNA, the effect of miR-21 was assessed on apoptosis of arsenite-transformed HELF cells. After arsenite-transformed cells were transfected with anti-miR-21, there were increases of cleaved-caspase-3 and decreases of caspase-3 levels (Figure 7A and 7B). In arsenite-transformed HELF cells, the rate of apoptosis of the anti-miR-21 group (16.81±1.50%) was higher than that of the anti-miR-nc group (3.56±0.89%) (Figure 7C). This infers that miR-21 has an essential role in the antiapoptotic capacity of these cells.

### Inhibition of miR-21 Decreases Cell Clonogenicity and Motility of Arsenite-transformed HELF Cells

**Figure 8:**
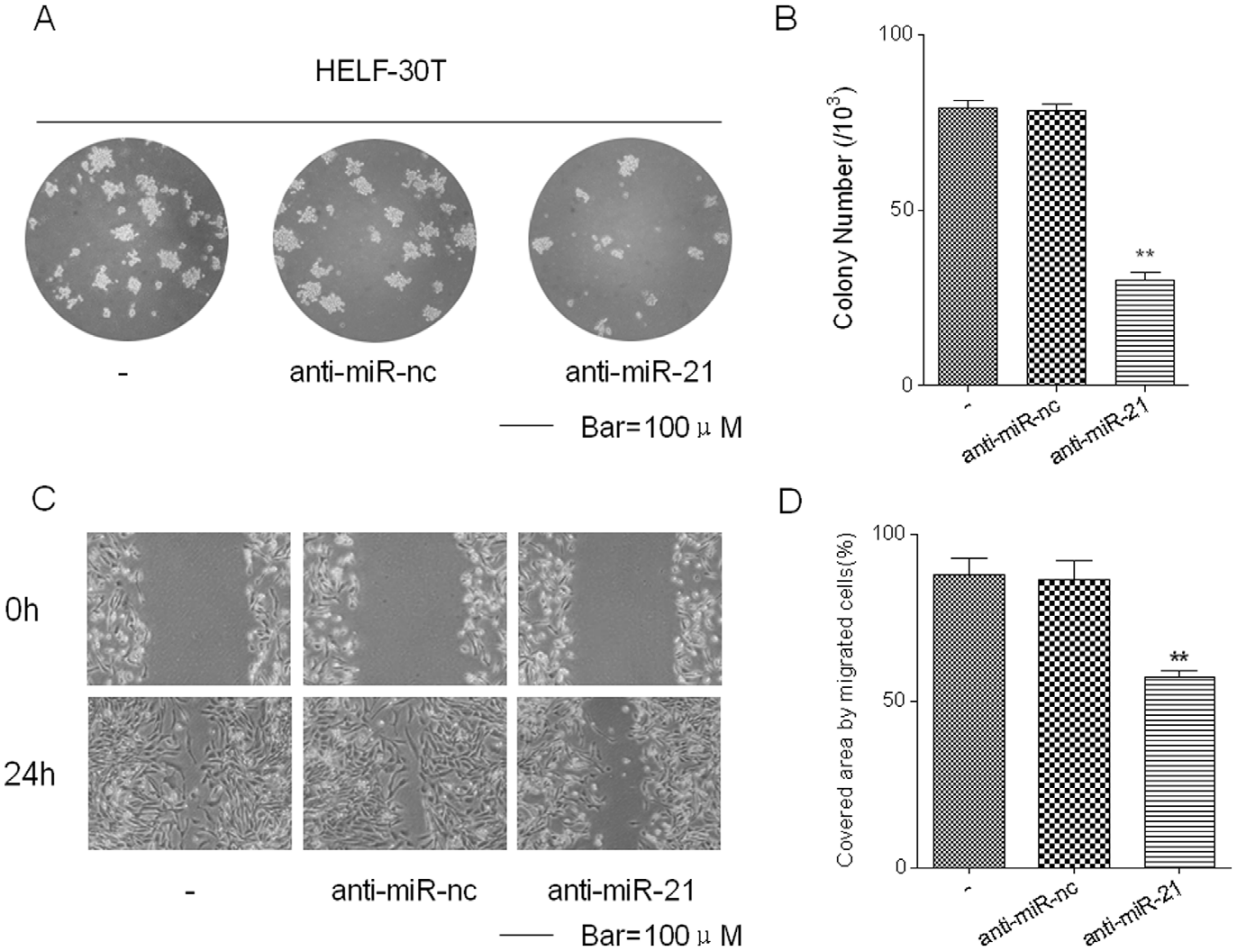
Inhibition of miR-21 decreases the neoplastic capacity and motility of arsenite-transformed HELF cells. After arsenite-transformed HELF cells were transfected with or without anti-miR-21 (150 nM) for 24 h, the neoplastic capacity and motility of cells were determined by anchorage-independent growth and scratch wound healing assays, respectively, bars = 100 µm. (A) Cell colony and (B) their numbers (means ± SD, n = 3) in soft agar. **P<0.01 difference from NC group. (C) Movement of cells into the wound and (D) the percentages of open space areas covered (means ± SD, n = 3). **P<0.01 difference from NC group.

The capacity for colony formation by arsenite-transformed HELF cells with or without transfection of anti-miR-21 was examined. The cells transfected with anti-miR-21 displayed fewer colonies (30±4) compared with the NC inhibitor group (78±3) (Figure 8A and 8B). An assay for wound-scratch healing, which is used to detect cell motility, was used to characterize the function of miR-21 on cell motility. Arsenite-transformed cells transfected with anti-miR-21 showed lower motility than cells transfected with anti-miR-nc (86.7±6.5%, in comparison with 0 h), and the relative rate was 59.5±7.7% (in comparison with 0 h) (Figure 8C and 8D). These results indicate that miR-21 is involved in cell clonogenicity and motility of arsenite-transformed HELF cells.

## Discussion

Arsenite is a well-established human carcinogen [5]. A close association and a positive correlation exist between arsenite exposure and increased incidence of various forms of cancer, as documented from studies in different arsenite-endemic areas in the world [40], [41]. Recently, the model of arsenite-induced malignant transformation has been widely used to investigate the mechanisms of arsenite-caused carcinogenesis, which remain obscure [2], [42], [43]. Several signal pathways and transcription factors, such as MAPKs, PI-3Ks, NF-κB, and c-Jun, leading to the alteration of gene expression responsible for cell growth, may be involved in arsenite-induced cell proliferation, malignant transformation, and apoptosis [3], [41], [44]. Here, with arsenite-transformed HELF cells, we observed the activation of JNK, ERK, Akt, NF-κB, and c-Jun. The results for passage-control HELF cells were similar to those for normal HELF cells, but different from those for arsenite-transformed HELF cells, which suggests that chronic exposure to arsenite induces the transformation of HELF cells.

MiRNAs may provide new insights for cancer research, including chemical carcinogenesis [45], [46]. Although progress has been made toward understanding the biogenesis and mechanisms of action of miRNAs, less is known about the effects of environmental exposures, especially to carcinogens such as arsenite, on miRNA expression. There are indications that individual miRNAs are involved in arsenite-induced malignant transformation [42]. MiR-21 is frequently over-expressed in human cancers [12], [47], and changes in miR-21 levels are associated with the development of tumors [8], [9], [14]. As shown in the present investigation, its expression was increased in cells transformed by arsenite, suggesting that miR-21 over-expression is associated with arsenite-induced malignant transformation.

MiR-21 is induced by two Ras downstream pathways, Ras-MAPKs and PI-3K [48], [49], [50]. It could be upregulated by RAS/ERK signaling through downregulating tumor suppressor RECK and thereby promoting malignant cell behavior [50]. In thyroid cells, the activation of Akt is also necessary to achieve miR-21 over-expression [48], [51]. As shown here, inhibition of JNK or ERK down-regulated the miR-21 expression level, but inhibition of PI-3K/Akt caused no change in the expression of miR-21. According to the other literature, activation of Akt is able to be promoted by the down-regulation of Pdcd4, Pten and Spry1, which are targets of miR-21 [29], [52]. In our study, we found the arsenite-induced sustained activations of Akt, therefore, we supposed that the regulation of miR-21 on Akt activation in HELF cells exposed to arsenite may probably through its targets. Besides, others have also indicated that control of miR-21 expression at the transcriptional level is regulated by STAT3 in human glioma cells as well as in myeloma and prostate cancer cells [53], [54]. Moreover, up-regulation of miR-21 expression through eliciting NF-κB recruitment to the miR-21 promoter region, where it cooperates with STAT3 to activate miR-21 transcription, has been verified [55].

MiRNA transcription, like that of mRNAs, is co-regulated by more than one transcription factor, either in cooperation or independently [56]. MiR-21 is regulated by the transcription factors, NF-κB and c-Jun [8], [57]. As c-Jun is activated through double phosphorylation by the JNK pathway, when JNK inhibitor (SP600125) was used to block the activation of JNK, the arsenite-induced activations of c-Jun and miR-21 were decreased. Since JNK was activated in arsenite-transformed HELF cells and miR-21 was up-regulated through phosphorylation of c-Jun, knockdown of c-Jun reduced the expression of miR-21. Further, activations of ERK and IKK are involved in NF-κB activation by arsenite in various cells [43]. When an ERK inhibitor (U0126) was used to block the activation of ERK in arsenite-transformed HELF cells, the expression levels of NF-κB p65 and miR-21 were decreased. Further, the blockade of NF-κB in these cells decreased the expression of miR-21. Therefore, we hypothesized that arsenite-induced up-regulation of miR-21 depends on the ERK and JNK pathways through activation of the transcription factors, NF-kB and c-Jun.

Based on the prediction of computer-aided algorithms, the tumor suppressor gene, *Pdcd4*, has been validated as a miR-21 target in human urothelial carcinoma and glioblastoma cells [14], [58]. In addition to being a suppressor of malignant transformation, tumorigenesis, and tumor progression, Pdcd4 is up-regulated in apoptosis and cellular senescence [13], [59]. Further, in colorectal cancers, miR-21 inhibits apoptosis and invasion by regulating Pdcd4 [60]. Based on the present results showing that miR-21 is a negative regulator of Pdcd4, we speculated that miR-21 has anti-apoptotic effects and inhibits invasion, at least in part, by negatively regulating Pdcd4. Since our transfection experiments showed that *Pdcd4* mRNA was unaltered, in contrast to changes in Pdcd4 protein, miR-21apparently induces Pdcd4 suppression by post-transcriptional control. Pdcd4 inhibits JNK activation by down-regulating MAP4K1, an upstream kinase of JNK [20], [21]. Moreover, c-Jun activity is essential for maintenance of the transformed phenotype, and inhibition of c-Jun activity by Pdcd4 suppresses the tumor cell phenotype [61]. Pdcd4 inhibits JNK1-dependent phosphorylation of c-Jun in a kinase reaction in vitro, an observation that supports our hypothesis [23].

The expression of different Spry family members is down-regulated in a variety of human cancers relative to normal adjacent tissue [62]. The modest activation of the ERK pathway seen upon Spry1 expression is enough to elicit senescence responses [30]. Moreover, our earlier studies have shown that, in HELF cells exposed to arsenite, inhibition of NF-kB prevents arsenite-induced decreases of Spry1, whereas Spry2 levels remain unchanged, and miR-21 regulates feedback on Spry1-mediated activation of ERK [13]. Indeed, miR-21 mediation of the down-regulation of its target gene, Sprys, a potent inhibitor of the Ras/MEK/ERK pathway, is essential for the maximal induction of ERK activity [8], [13], [31].

Thus, we hypothesized that JNK and ERK are regulated by feedback of the miR-21 target genes, Pdcd4 and Spry1. We demonstrated that, for arsenite-transformed HELF cells, knockdown of miR-21 decreases the phosphorylation of JNK and ERK, which is due to increased protein levels of Pdcd4 and Spry1, respectively. Arsenite-induced up-regulation of miR-21 was dependent on ERK and JNK through activation of transcription factors, NF-κB and c-Jun, respectively, and is involved in malignant transformation. Further, miR-21 sustained continuing activation of MAPKs/JNK/ERK.

Apoptosis is a process that is dysregulated in tumorigenesis [63]. Since activation of caspases is a marker of apoptosis, the expression of cleaved caspase-3, a key facilitator of apoptosis, was determined. Its protein levels were increased when miR-21 was knocked down. The malignancy potential of tumor cells is reflected by their degree of anchorage-independent growth [64]. Thus, arsenite-transformed HELF cells transfected with anti-miR-21 displayed fewer colonies. Cell clonogenicity and motility reflect the invasive capacity of cells. Invasiveness is a characteristic of malignancies, and is involved in tumor metastasis and recurrence, presenting a difficult challenge for tumor therapy and prognosis [64], [65]. Arsenite-transformed HELF cells transfected with anti-miR-21 showed a lower motility than cells transfected with anti-miR-nc. MiR-21 was over-expressed in arsenite-transformed HELF cells, and inhibition of miR-21 resulted in increases in apoptosis, decreases in clonogenicity, and reductions in motility of these cells, which suggests that miR-21 up-regulation is a key step leading to oncogenesis, malignancy, and metastasis of arsenite-transformed HELF cells.

**Figure 9:**
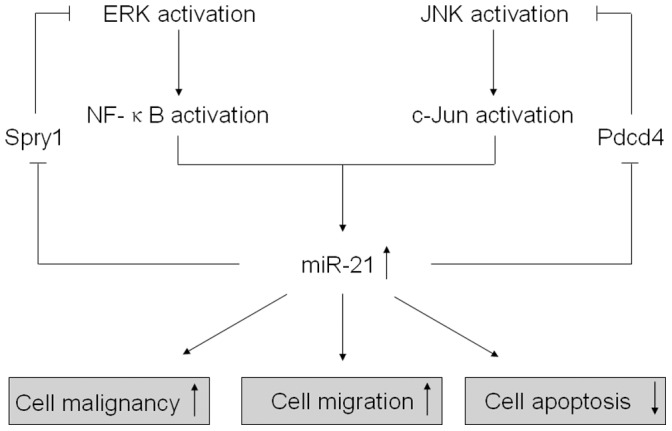
Feedback regulations of miR-21 and MAPKs via Pdcd4 and Spry1 in arsenite-induced malignant transformation of HELF cells. In arsenite-transformed HELF cells, miR-21 levels are up-regulated by the activations of ERK/NF-κB and JNK/c-Jun. miR-21 inhibits the target proteins, Pdcd4 and Spry1, which form two feedback regulation loops, miR-21-Spry1-ERK/NF-κB and miR-21-Pdcd4-JNK/c-Jun.

We have previously demonstrated the regulation of miR-21 by ROS-activated ERK/NF-kB in arsenite-induced cell transformation [13]. In the present study, miR-21 levels were up-regulated by the activations of ERK/NF-κB and JNK/c-Jun, and miR-21 inhibited target proteins of Pdcd4 and Spry1, which form two feedback regulation loops of miR-21-Spry1-ERK/NF-κB and miR-21-Pdcd4-JNK/c-Jun in arsenite-transformed HELF cells (Figure 9). Our previous study mainly stressed that the expression of miR-21 is involved in the process of cell transformation by arsenite. Results in the present report, however, are more systematic and present the roles of feedback regulations of miR-21 and MAPKs via Pdcd4 and Spry1 in arsenite-induced malignant transformation of HELF cells. The results point to the function of feedback regulations of miR-21 and MAPKs in the later period of arsenite-transformed cells, which are necessary for maintenance of transformation and malignant progression. The results of the present study are in agreement with those of our previous study, which suggested that our model of malignant transformation is appropriate, and that the results are reproducible. Thus, the present study expands knowledge of the mechanisms of arsenite-induced transformation regulated by feedback of ERK/miR-21/Spry1.

These results indicate that the feedback regulations of miR-21 and MAPKs via Pdcd4 and Spry1 are involved in arsenite-induced carcinogenesis and in promotion of cell malignancy, migration, and apoptosis. They enhance our understanding of arsenite-induced carcinogenesis and establish that miR-21 serves as an important target in developing treatments for carcinogenesis induced by environmental pollutants.

## Supporting Information

Figure S1 **Activations of ERK/NF-κB, JNK/c-Jun, and Akt are induced in normal HELF cells by a low level of arsenite.** Densities of bands were quantified by Eagle Eye II software. GAPDH levels, measured in parallel, served as controls. Normal HELF cells were exposed to 1.0 µM arsenite for 0, 6, or 24 h. (A, B) Western blot analyses and (C) relative protein levels (means ± SD, n = 3) of p-ERK, p-JNK, and p-p38 (representative of MAPKs signal pathways); levels of p- NF-κB 65 and p-c-Jun (representative transcription factors); and level of p-Akt (representative of the PI-3Ks signal pathway). **P<0.01 different from HELF cells exposed to arsenite for 0 h.
(TIF)

Figure S2 **The level of miR-21 is up-regulated, and the protein levels of Pdcd4 and Spry1 are decreased in normal HELF cells by a low level of arsenite.** Densities of bands were quantified by Eagle Eye II software. GAPDH levels, measured in parallel, served as controls. Normal HELF cells were exposed to 1.0 µM arsenite for 0, 6, or 24 h. (A) The levels of miR-21 were determined by qRT-PCR assays (means ± SD, n = 3). **P<0.01 different from normal HELF cells exposed to arsenite for 0 h. (B) The protein levels (upper) and mRNA levels (lower) of Pdcd4 and Spry1 (target proteins of miR-21) were analyzed by Western blots and RT-PCR, respectively. (C) The relative protein levels of Pdcd4 and Spry1 (means ± SD, n = 3). **P<0.01 different from normal HELF cells exposed to arsenite for 0 h.
(TIF)
