# Supplementary figures and images for: Feedback Regulations of miR-21 and MAPKs via Pdcd4 and Spry1 Are Involved in Arsenite-Induced Cell Malignant Transformation
Source: PLoS One. 2013 Mar 1;8(3):e57652. doi: 10.1371/journal.pone.0057652 (PMC3585869; doi:10.1371/journal.pone.0057652)

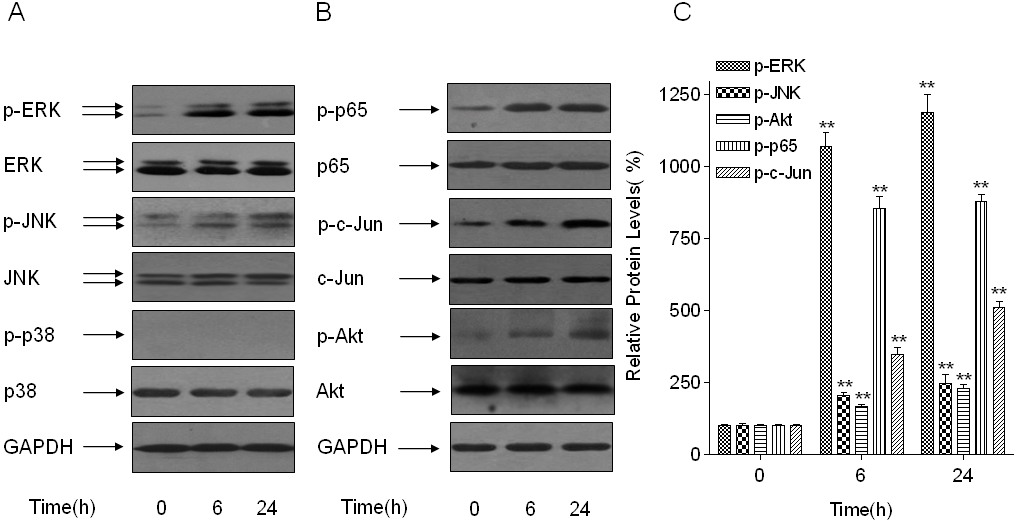

Supplement: Figure S1 — Activations of ERK/NF-κB, JNK/c-Jun, and Akt are induced in normal HELF cells by a low level of arsenite. Densities of bands were quantified by Eagle Eye II software. GAPDH levels, measured in parallel, served as controls. Normal HELF cells were exposed to 1.0 µM arsenite for 0, 6, or 24 h. (A, B) Western blot analyses and (C) relative protein levels (means ± SD, n = 3) of p-ERK, p-JNK, and p-p38 (representative of MAPKs signal pathways); levels of p- NF-κB 65 and p-c-Jun (representative transcription factors); and level of p-Akt (representative of the PI-3Ks signal pathway). **P<0.01 different from HELF cells exposed to arsenite for 0 h. (TIF) [file pone.0057652.s001.tif]

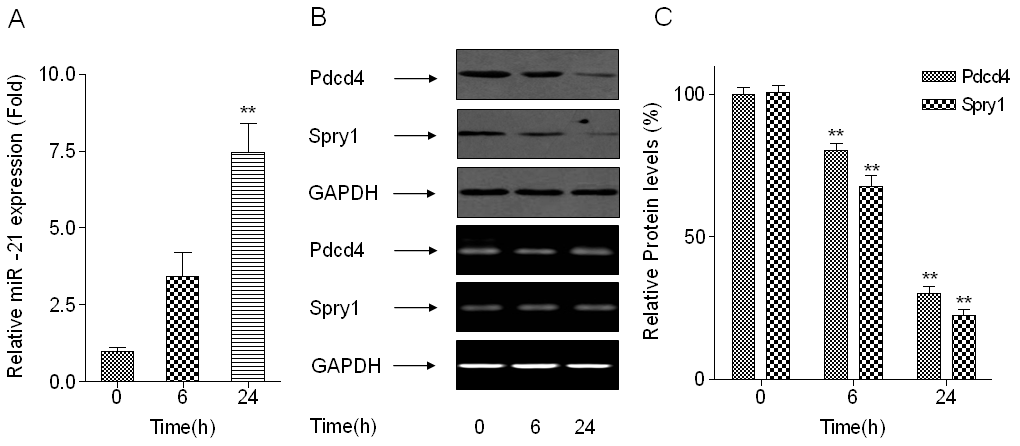

Supplement: Figure S2 — The level of miR-21 is up-regulated, and the protein levels of Pdcd4 and Spry1 are decreased in normal HELF cells by a low level of arsenite. Densities of bands were quantified by Eagle Eye II software. GAPDH levels, measured in parallel, served as controls. Normal HELF cells were exposed to 1.0 µM arsenite for 0, 6, or 24 h. (A) The levels of miR-21 were determined by qRT-PCR assays (means ± SD, n = 3). **P<0.01 different from normal HELF cells exposed to arsenite for 0 h. (B) The protein levels (upper) and mRNA levels (lower) of Pdcd4 and Spry1 (target proteins of miR-21) were analyzed by Western blots and RT-PCR, respectively. (C) The relative protein levels of Pdcd4 and Spry1 (means ± SD, n = 3). **P<0.01 different from normal HELF cells exposed to arsenite for 0 h. (TIF) [file pone.0057652.s002.tif]
